# Comparative Evaluation of Recent Tests for Assessing Craving in Alcohol Use Disorder

**DOI:** 10.1192/j.eurpsy.2026.10880

**Published:** 2026-07-31

**Authors:** G. M. Festa, G. Martinotti, I. S. Lancia, A. Viggiani, C. Busdraghi

**Affiliations:** 1 Pontifical Faculty of Educational Sciences «AUXILIUM»; 2 Interdisciplinary Institute of Advanced Clinical Training «IACT», Rome; 3 University G.d’Annunzio , Chieti-Pescara; 4 San Carlo Regional Hospital, Potenza; 5 Giunti Psychometrics, Florence, Italy

## Abstract

**Introduction:**

The identification and quantification of craving in Alcohol Use Disorder (AUD) are fundamental for both clinical practice and research. The various tools available across countries and in the literature raise issues of selection, reliability and comparability across contexts and populations.

**Objectives:**

This study focused on identifying various assessment tools (inclusion criteria: tests published from 2020 to the present) for evaluating and measuring alcohol craving. The aim was to provide a critical review of these tools, with a focus on their structure and psychometric properties.

**Methods:**

7 tests were identified:

Approach and Avoidance of Alcohol Questionnaire (AAAQ)

Alcohol Craving Experience (ACE)

Alcohol Craving Questionnaire (ACQ)

Alcohol Craving Scale Based on Three Factors (ACS-3F)

Alcohol Craving Typology Questionnaire (A-CTQ)

Adolescent-Obsessive Compulsive Drinking Scale (A-OCDS)

Jellinek Craving Questionnaire (JACQ)

Multidimensional Alcohol Craving Scale (MACS)

The aspects were analyzed: year of publication, structure, content, reliability, standardization sample, and administration method.

**Results:**

All identified tools showed good internal consistency (Cronbach’s α > 0.70).

**AAAQ** was designed to assess two aspects of alcohol craving: the desire to drink and the inclination to avoid alcohol consumption.

**ACE** measures sensory and cognitive aspects of craving, such as imagining the taste, smell, or sensations of drinking, as well as intrusive thoughts about alcohol. It identifies three main factors: imagination, intensity, intrusion.

**ACS-3F** assesses responses to positively reinforcing stimuli, negatively reinforcing stimuli, and loss of control.

**A-CTQ** is the most recent scale among those reviewed and includes a large normative and clinical standardization sample. It distinguishes accurately between different types of craving (Reward, Relief, Obsessive), with specific therapeutic implications.

**A-OCDS** is suitable for adolescents and young adults. It assesses the frequency of alcohol-related thoughts, desires, and imagery, difficulties in avoiding alcohol, and tendencies to skip or drink before/during school hours.

**JACQ** evaluates craving across four dimensions: emotional impulse, physical sensations, temptation to drink, and uncontrolled thoughts. It is structured to assess both current (immediate urge) and past (retrospective urge) craving.

**MACS** specifically measures two main dimensions: alcohol desire and behavioral disinhibition. It evaluates craving intensity and presence while considering aspects of alcohol-use planning and anticipation of positive effects or abstinence improvement.

**Image 1:**
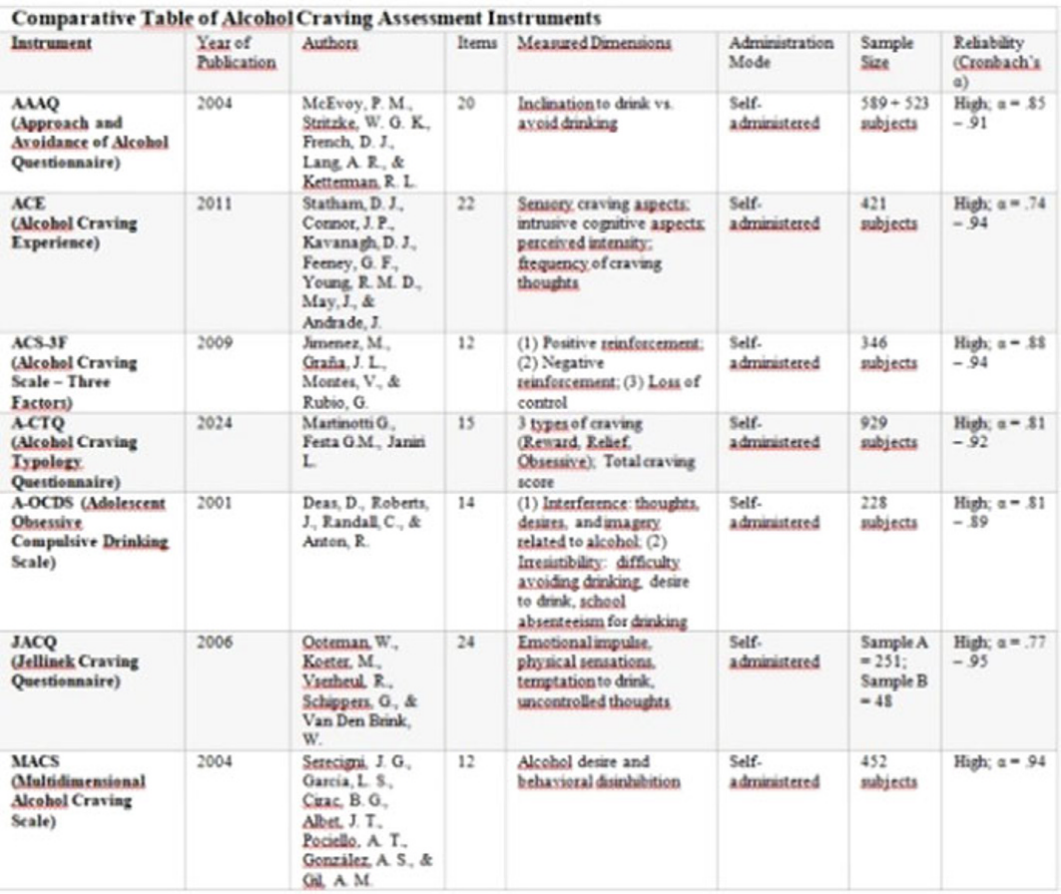
Image 1: Long description.

**Image 2:**
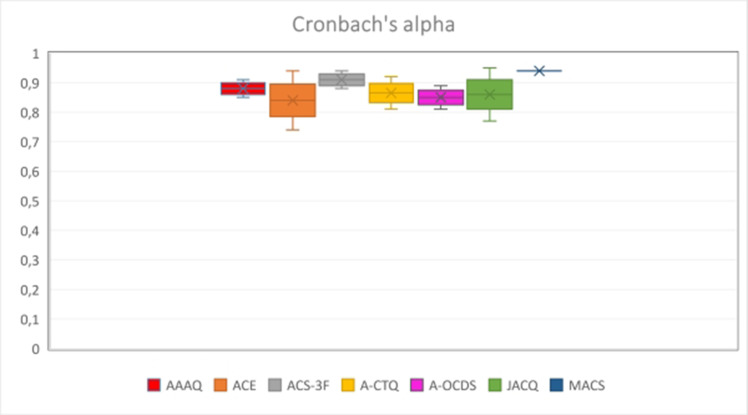
Image 2: Long description.

**Image 3:**
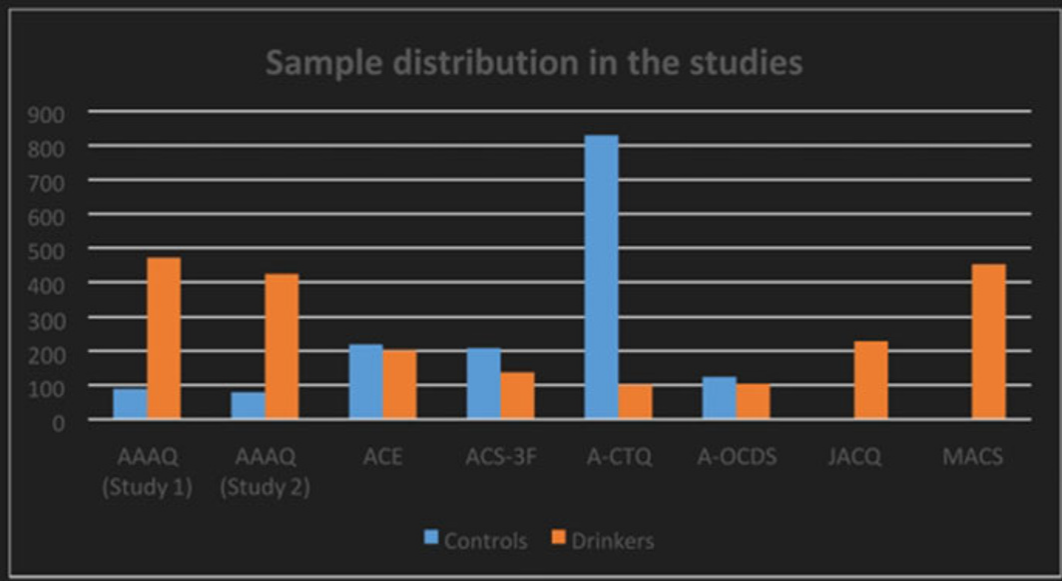
Image 3: Long description.

**Conclusions:**

The choice of instrument in craving assessment depends on the goal (screening, monitoring, classification), the context (clinical vs. research, patient age), the target population, and the available time for assessment. All the tools reviewed represent valid aids for clinical work in the field of alcohol use disorder.

**Disclosure of Interest:**

None Declared

## Long descriptions

### Image 1: Long description

The table titled Comparative Table of Alcohol Craving Assessment Instruments contains eight columns: Instrument, Year of Publication, Authors, Items, Measured Dimensions, Administration Mode, Sample Size, and Reliability (Cronbach’s alpha).

* A A A Q (Approach and Avoidance of Alcohol Questionnaire): 2004; McEvoy et al.; 20 items; Inclination to drink vs. avoid drinking; Self-administered; 589 + 523 subjects; High alpha = .85 to .91.

* A C E (Alcohol Craving Experience): 2011; Statham et al.; 22 items; Sensory craving aspects, intrusive cognitive aspects, perceived intensity, frequency of craving thoughts; Self-administered; 421 subjects; High alpha = .74 to .94.

* A C S 3 F (Alcohol Craving Scale Three Factors): 2009; Jimenez et al.; 12 items; 1 Positive reinforcement, 2 Negative reinforcement, 3 Loss of control; Self-administered; 346 subjects; High alpha = .88 to .94.

* A C T Q (Alcohol Craving Typology Questionnaire): 2024; Martinotti et al.; 15 items; 3 types of craving (Reward, Relief, Obsessive), Total craving score; Self-administered; 929 subjects; High alpha = .81 to .92.

* A O C D S (Adolescent Obsessive Compulsive Drinking Scale): 2001; Deas et al.; 14 items; 1 Interference: thoughts, desires, and imagery related to alcohol, 2 Irresistibility: difficulty avoiding drinking, desire to drink, school absenteeism for drinking; Self-administered; 228 subjects; High alpha = .81 to .89.

* J A C Q (Jellinek Craving Questionnaire): 2006; Ooteman et al.; 24 items; Emotional impulse, physical sensations, temptation to drink, uncontrolled thoughts; Self-administered; Sample A = 251, Sample B = 48; High alpha = .77 to .95.

* M A C S (Multidimensional Alcohol Craving Scale): 2004; Serecigni et al.; 12 items; Alcohol desire and behavioral disinhibition; Self-administered; 452 subjects; High alpha = .94.

Navigate back to Image 1:.

### Image 2: Long description

The y-axis represents Cronbach's alpha values ranging from 0 to 1 in increments of 0.1. The x-axis lists seven categories with corresponding colored box plots.

From left to right, the data points are

* A A A Q (red): Box centered around 0.88 with narrow whiskers.

* A C E (orange): The widest range, with a median near 0.84 and whiskers extending from approximately 0.74 to 0.94.

* A C S-3 F (grey): High reliability with a median above 0.9 and very tight whiskers.

* A-C T Q (yellow): Median around 0.87 with whiskers between 0.81 and 0.92.

* A-O C D S (pink): Median near 0.85 with whiskers between 0.81 and 0.89.

* J A C Q (green): Median near 0.86 with whiskers between 0.77 and 0.95.

* M A C S (dark blue): Represented as a single point with a horizontal error bar at approximately 0.94.

All boxes are positioned in the upper quadrant of the graph, indicating high internal consistency across all measured scales.

Navigate back to Image 2:.

### Image 3: Long description

The y-axis represents sample size from 0 to 900 in increments of 100. The x-axis lists eight categories. A legend at the bottom identifies blue bars as Controls and orange bars as Drinkers.

* A A A Q (Study 1) has approximately 100 Controls and 480 Drinkers.

* A A A Q (Study 2) has approximately 80 Controls and 420 Drinkers.

* A C E has approximately 220 Controls and 200 Drinkers.

* A C S-3 F has approximately 210 Controls and 140 Drinkers.

* A-C T Q shows a significant peak for Controls at approximately 830, with Drinkers at approximately 100.

* A-O C D S has approximately 130 Controls and 100 Drinkers.

* J A C Q has 0 Controls and approximately 230 Drinkers.

* MACS has 0 Controls and approximately 450 Drinkers.

Navigate back to Image 3:.

